# Comment on Kumarasamy et al. Prognostic Utility of Platelet–Lymphocyte Ratio, Neutrophil–Lymphocyte Ratio and Monocyte–Lymphocyte Ratio in Head and Neck Cancers: A Detailed PRISMA Compliant Systematic Review and Meta-Analysis. *Cancers* 2021, *13*, 4166

**DOI:** 10.3390/cancers17243919

**Published:** 2025-12-08

**Authors:** Dimitris Tatsis, Alexandros Louizakis, Angeliki Cheva

**Affiliations:** 1 Department of Oral and Maxillofacial Surgery, Aristotle University of Thessaloniki, 54124 Thessaloniki, Greece; dtatsis@outlook.com; 2 Pathology Department, Faculty of Medicine, Aristotle University of Thessaloniki, 54124 Thessaloniki, Greece; antacheva@yahoo.gr

Exploring minimally invasive prognostic markers in head and neck squamous cell carcinoma is of value. To be robust and applicable research must adhere to strict inclusion criteria. This 2021 study on the prognostic utility of platelet–lymphocyte ratio (PLR), neutrophil–lymphocyte ratio (NLR), and monocyte–lymphocyte ratio (MLR) in head and neck cancers (HNCs), however, included esophageal, gastric, and thyroid cancers in HNCs [1]. These should not be grouped with HNCs, nor should lip cancers. Esophageal cancers often encompass both SCC and adenocarcinoma; Barrett’s esophagus and gastroesophageal reflux disease (GERD) are precursors for adenocarcinoma [2,3,4]. Gastric adenocarcinomas are influenced by Helicobacter pylori infection [5,6]. Thyroid carcinomas are also obviously different. The inclusion of studies on esophageal and gastric cancers in a meta-analysis aimed at elucidating HNC prognostic markers, introduces unwarranted unrelated heterogeneity. This is compounded by the study’s reported high between-study heterogeneity (e.g., I^2^ > 50% in pooled analyses), which may obscure conclusions about the prognostic relevance of PLR, NLR, and MLR [6].

Peripheral blood inflammatory biomarkers (PLR, NLR, MLR) may provide prognostic utility in HNCs. NLR had the highest accuracy (81%) in detecting occult neck metastases in early-stage oral cavity carcinomas (OCCs), but MLR lacks statistical significance (*p* = 0.73) [7]. Elevated NLR was reported as a negative prognostic factor in 40 cases of head and neck squamous cell carcinoma (SCC) [8]. 

Among HNCs, there should be distinction among EBV^+^ nasopharyngeal carcinoma, HPV^+^ oropharyngeal carcinoma, and non-virus-related SCC (e.g., in oral cavity and larynx). That these tumors are anatomically nearby is not a reason to group them together.

We strongly advocate for more focused investigations to delineate the role of these markers.
